# Engineering and Validation of a Vector for Concomitant Expression of Rare Transfer RNA (tRNA) and HIV-1 *nef* Genes in *Escherichia coli*

**DOI:** 10.1371/journal.pone.0130446

**Published:** 2015-07-06

**Authors:** Siti Aisyah Mualif, Sin-Yeang Teow, Tasyriq Che Omar, Yik Wei Chew, Narazah Mohd Yusoff, Syed A. Ali

**Affiliations:** 1 Oncological and Radiological Sciences, Advanced Medical and Dental Institute, Universiti Sains Malaysia, Pulau Pinang, Malaysia; 2 Regenerative Medicine, Advanced Medical and Dental Institute, Universiti Sains Malaysia, Pulau Pinang, Malaysia; New England BioLabs, UNITED STATES

## Abstract

Relative ease in handling and manipulation of *Escherichia coli* strains make them primary candidate to express proteins heterologously. Overexpression of heterologous genes that contain codons infrequently used by *E*. *coli* is related with difficulties such as mRNA instability, early termination of transcription and/or translation, deletions and/or misincorporation, and cell growth inhibition. These codon bias -associated problems are addressed by co-expressing ColE1-compatible, rare tRNA expressing helper plasmids. However, this approach has inadequacies, which we have addressed by engineering an expression vector that concomitantly expresses the heterologous protein of interest, and rare tRNA genes in *E*. *coli*. The expression vector contains three (*arg*U, *ile*Y, *leu*W) rare tRNA genes and a useful multiple cloning site for easy in-frame cloning. To maintain the overall size of the parental plasmid vector, the rare tRNA genes replaced the non-essential DNA segments in the vector. The cloned gene is expressed under the control of T7 promoter and resulting recombinant protein has a C-terminal 6His tag for IMAC-mediated purification. We have evaluated the usefulness of this expression vector by expressing three HIV-1 genes namely HIV-1 p27 (*nef*), HIV-1 p24 (*ca*), and HIV-1 *vif* in NiCo21(DE3) *E*.*coli* and demonstrated the advantages of using expression vector that concomitantly expresses rare tRNA and heterologous genes.

## Introduction

Bacterium *Escherichia coli* (*E*. *coli*) remains a predominant host for the expression of heterologous proteins. Like other organisms, *E*. *coli* uses 61 available amino acid codons for mRNA production. However, not all 61 mRNA codons are used equally [1, 2]. The so-called ‘major’ codons occur in highly expressed genes, whereas ‘rare’ codons are present in low expressing genes [1, 2, 3]. The frequency of the codon usage in mRNAs also reflects the abundance of their cognate tRNAs in the cells. When the codon usage of the overexpressed heterologous protein differs considerably from the normal codon usage of the expression host, protein synthesis can be inhibited due to the depletion of rare tRNAs cellular pool [4].

Viral proteins are encoded by genes that contain codons rarely used by *E*. *coli*. For instance, genes of HIV-1 proteins contain 8.21% (in gene encoding Nef protein) up to 23.17% (in gene encoding Vpu protein) codons that are rarely used by *E*. *coli* (**Table 1**). These genes express poorly in *E*. *coli* and as a result, little and/or poor quality protein is produced [4, 5]. To alleviate codon bias-associated problems, one option is to optimize the gene sequence by changing rare codons into more frequently used codons [6]. Alternatively, specialized *E*. *coli* strains such as BL21-CodonPlus (Stratagene) and Rosetta2(DE3) (EMD Millipore) can be used. These strains harbor ColE1-compatible, rare tRNA expressing helper plasmids, which are maintained under chloramphenicol selective pressure [7].

**Table 1:** Rarely used codons by *E*. *coli* in genes of HIV-1.

HIV-1 proteins	Total number of codons per ORF	Number of codons rarely used by *E*. *coli*	Percentage of codons rarely used by *E*. *coli*
Gag polyprotein	501	55	10.97
Pol poly protein	1004	101	10.05
Vif	193	28	14.5
Vpr	97	17	17.52
Tat	87	11	12.64
Rev	117	19	16.24
Vpu	82	19	23.17
Env	855	95	11.11
Nef	207	17	8.21

HIV-1 (NL4-3) genes were subjected to rare codon analysis using online tool ‘*Rare Codon Calculator (RaCC)*’ http://nihserver.mbi.ucla.edu/RACC/.

High level expression of numerous heterologous proteins has been achieved by using either of the two above mentioned strategies. However, there are some issues associated with these approaches. **1.** Codon optimization via gene synthesis can be costly and time-consuming especially for genes longer than 500bp. Moreover, codon changes can affect secondary structure of mRNA with unknown consequences [8, 9]. **2**. Maintenance of tRNA-expressing helper plasmids together with expression vectors results in additional metabolic pressure because the bacteria constitutively express two antibiotic resistance genes [10]. **3**. It complicates expression strategies where multiple vectors are employed for co-expression of protein subunits [11]. **4**. Certain engineered strains such as those containing pLysS (to reduce background expression levels) cannot be transformed with rare tRNA vectors that contain p15A ori and constitutively express chloramphenicol acetyltransferase gene for selection [12].

To address abovementioned limitations, we engineered an expression vector that would express both the heterologous protein of interest, and rare tRNA genes in *E*. *coli*. We started off with cloning HIV-1 *nef* gene in an expression vector pSA-HP24-6His, which we have previously used for high level expression of HIV-1 *p24* [13]. We expressed HIV-1 Nef because it has gained increased interest as a new therapeutic target for HIV/AIDS treatment in recent years [14, 15, 16, 17, 18, 19] and we are engineering cell internalizing antibodies to target this pathogenic factor. We then modified the backbone of the resulting pSA-HNef-6His vector by replacing a non-essential DNA segment between *lac*I gene and T7 promoter with rare tRNA genes *arg*U, *ile*Y, and *leu*W. We call this vector pSA-HNef-6His-RIL. In order to further validate the utility of this rare tRNA gene vector, we replaced *nef* with HIV-1 *p24* and *vif* genes and the resulting plasmids were designated as pSA-HP24-6His-RIL and pSA-HVif-6His-RIL respectively, which were then used to express P24 and Vif proteins. To facilitate clone manipulation, we substituted the *nef* gene with a multiple cloning site (MCS) and the resulting plasmids called pSA-C6His-RIL.

## Materials and Methods

### Bacterial culture conditions

The *E*. *coli* strains DH5α (NEB, #C2987H) and NiCo21(DE3) (NEB, #C2529H) were used for cloning and expression experiments, respectively. Bacteria were grown aerobically in LB (Miller) broth, or on LB (Miller) agar at different temperatures, and in the presence or absence of ampicillin (100μg/ml) and/or chloramphenicol (25μg/ml). Bacterial strains were stored in glycerol (50%)-supplemented LB broth at -80°C. In some experiments, NiCo21(DE3) were transformed with rare tRNA expressing pACYC-RIL (Stratagene), pRARE2 (Novagen), and pLysSRARE2 (Novagen) plasmids and the transformants were selected on chloramphenicol-supplemented LB-agar plates.

### Plasmid construction

PCR amplifications of DNA fragments, intended for cloning purposes, was carried out using Q5 High-Fidelity DNA Polymerase (NEB, #M0491), whereas Taq DNA Polymerase (NEB, #M0273) was used for colony PCR. DNA fragments were reaction cleaned-up or gel-purified using NucleoSpin Gel and PCR Clean-up kit (Macherey-Nagel GmbH & Co, #740609). Plasmid DNA was purified using Wizard Plus SV Minipreps DNA Purification System (Promega, #A1465). Vector and insert were mixed in 1:3 molar ratios (unless otherwise specified) and ligated in presence of T4 DNA ligase (NEB, #M0202) at 4°C for 18 h. Construction of engineered vectors (**Table 2**) is described below.

**Table 2:** Expression vectors engineered in this study.

Vector name	Features
pSA-HNef-6His (6.517kb)	a. A derivative of pSA-Hp24-6His [13]
	b. Expresses HIV-1 (NL4-3) Nef protein under the control of T7 promoter.
	c. Contains a 6His tag on C-terminal of HIV-1 Nef.
pSA-HNef-6His-RIL (6.455kb)	a. A derivative of pSA-HNef-6His.
	b. Expresses *arg*U, *ile*Y, and *leu*W tRNA genes under the control of their own promoters.
pSA-HP24-6His-RIL (6.533kb)	a. A derivative of pSA-HNef-6His-RIL.
	b. Expressed HIV-1 (NL4-3) P24 protein under the control of T7 promoter.
pSA-HVif-6His (6.500kb)	a. A derivative of pSA-HNef-6His.
	b. Expresses HIV-1 (NL4-3) Vif protein under the control of T7 promoter.
pSA-HVif-6His-RIL (6.439kb)	a. A derivative of pSA-HNef-6His-RIL
	b. Expresses HIV-1 (NL4-3) Vif protein under the control of T7 promoter.
pSA-C6His-RIL (5.885kb)	a. A derivative of pSA-HNef-6His-RIL.
	b. HIV-1 Nef gene is replaced with a multiple cloning site (MCS).

### Construction of pSA-HNef-6His

The HIV-1 *nef* gene encoding 206 residues of wild type Nef protein was PCR amplified from pNL4.3 plasmid (NIH AIDS Reagent Program, #114) using Nef-*Nde*I-F and Nef-*Sac*I-R primers (**Table 3**). The resulting 635bp amplicon was gel purified and restricted with *Nde*I (NEB, #R0111L) and *Sac*I (NEB, #R0156L) for 8h at 37°C, and purified. The vector was prepared by restricting pSA-HP24-6His [13] with *Nde*I/*Sac*I for 4h., separating plasmid backbone on 1% agarose gel, and purified. Insert (635bp *nef*) and vector (5.914kb pSA-6His) were ligated, transformed into chemically competent DH5α *E*. *coli* cells, and selected on ampicillin-containing LB-agar plates after 18h incubation at 30°C. Ten randomly selected bacterial colonies were subjected to colony PCR using vector-specific pMXB10-up101-F and insert-specific Nef-R primers (**Table 3**). Transformants that contained an amplicon of expected size by PCR were then verified using DNA restriction and sequence analyses. This vector was called pSA-HNef-6His.

**Table 3:** Oligonucleotides used to construct and verify pSA-HNef-6His, pSA-HNef-6His-RIL, pSA-Hp24-6His-RIL, pSA-HVif-6His-RIL, and pSA-C6His-RIL expression vectors.

Primer	Sequence (5’ to 3’)
Nef-*Nde*I-F	GGTGGT*CATATG*GGTGGCAAGTGGTCAAAAAG
Nef-*Sac*I-F	GGTGGT*GAGCTC*GCAGTTCTTGAAGTACTCCGG
Up-T7-*Sma*I-F	TCAGA*CCCGGG*GCCAGGAATTGGGGATCGG
Up-lacI-*Sma*I-R	TCAGA*CCCGGG*GCATGCACCATTCCTTGCG
pMXB10-up101-F	GATCCCGCGAAATTAATACG
Nef-R	GCAGTTCTTGAAGTACTCCGG
pMXB4560-81-Seq-F	CTCCTGCATTAGGAAGCAGCCC
RIL-R	CCCATCCGTACAACGCTTTC
P24-*Nde*I-F	GGTGGTCATATGCCTATAGTGCAGAACCTCCAG
P24-*Sac*I-R	GGTGGTGAGCTCCAAAACTCTTGCTTTATGGCC
P24-R	CAAAACTCTTGCTTTATGGCC
Vif-*Nhe*I-F	GTGGTGGCTAGCATGGAAAACAGATGGCAGGTG
Vif-*Sac*I-R	CCTTAAGAGCTCGTGTCCATTCATTGTATGGCTCC
Vif-R	GTGTCCATTCATTGTATGGCTCC
pSA-*Nhe*I-R	CATCATGCTAGCGTATATCTCCTTCTTAAAGTTAAAC
pSA-F	CAAGAACTGCGAGCTCCAC
MCS-F	TATGAGATCTGCTAGCGAATTCTCGCGACCGCGGGTCGACGCGGCCGCAGAGCT
MCS-R	CTGCGGCCGCGTCGACCCGCGGTCGCGAGAATTCGCTAGCAGATCTCA

The restriction sites are in italics.

### Construction of pSA-HNef-6His-RIL

The pACYC-RIL vector (5μg) was restricted with *Ssp*I (NEB, #R0132) and *Fsp*I (NEB, #R0135) for 4h at 37°C, and an 874bp DNA fragment that contained *arg*U, *ile*Y, *leu*W tRNA genes, was purified from 1.5% agarose gel. The vector was prepared by PCR amplifying 100ng of pSA-HNef-6His using UpT7-*Sma*I-F and Up*lac*I-*Sma*I-R primers (**Table 3**) for 25 cycles. The resulting 5.581kb PCR-amplified vector backbone was reaction cleaned-up and restricted with *Sma*I (NEB, #R0141) for 8h at 30°C. Ten units of *Dpn*I (NEB, #R0176) and 1 unit of Shrimp Alkaline Phosphatase (NEB, #M0371) was added to the same reaction and incubated for 60min at 37°C. The vector backbone was then purified from 1% agarose gel. Insert (874bp *arg*U, *ile*Y, *leu*W-containing DNA) and vector (5.581kb pSA-HNef-6His) were ligated, transformed into chemically competent DH5α *E*. *coli* cells, and selected on ampicillin-containing LB-agar plates after 18h incubation at 30°C. Ten randomly selected bacterial colonies were subjected to colony PCR using vector-specific pMXB4560-81Seq-F and insert-specific RIL-R primers (**Table 3**). Transformants that contained expected size of amplicon by PCR were then verified using DNA restriction and sequence analyses.

### Construction of pSA-HP24-6His-RIL

The HIV-1 *p24* gene encoding 232 residues of wild type P24 protein was PCR amplified from pNL4.3 plasmid (NIH AIDS Reagent Program, #114) using P24-*Nde*I-F and P24-*Sac*I-R primers (**Table 3**). The resulting 713bp amplicon was gel purified and restricted with *Nde*I (NEB, #R0111L) and *Sac*I (NEB, #R0156L) for 8h at 37°C, and purified. The vector was prepared by restricting pSA-HNef-6His-RIL vector (5μg) with *Nde*I (NEB, #R0111L) and *Sac*I (NEB, #R0156L) for 8h at 37°C, and purified. Insert (702bp *p24*) and vector (5.831kb pSA-6His-RIL) were ligated, transformed into chemically competent DH5α *E*. *coli* cells, and selected on ampicillin-containing LB-agar plates after 18h incubation at 30°C. Ten randomly selected bacterial colonies were subjected to colony PCR using vector-specific pMXB10-up101-F and insert-specific P24-R primers (**Table 3**). Transformants that contained an amplicon of expected size by PCR were then verified using DNA restriction and sequence analyses.

### Construction of pSA-HVif-6His and pSA-HVif-6His-RIL

The HIV-1 *vif* gene encoding 192 residues of wild type Vif protein was PCR amplified from pNL4.3 plasmid (NIH AIDS Reagent Program, #114) using Vif-*Nhe*I-F and Vif-*Sac*I-R primers (**Table 3**). The resulting 600bp amplicon was gel purified and restricted with *Nhe*I (NEB, #R0131L) and *Sac*I (NEB, #R0156L) for 8h at 37°C, and purified. Two vector backbones were prepared by whole plasmid PCR of 100 ng pSA-HNef-6His and pSA-HNef-6His-RIL using pSA-*Nhe*I-R and pSA-F primers (**Table 3**) and 25 cycles. The resulting 5.914kb (pSA-6His) and 5.853kb (pSA-6His-RIL) PCR-amplified vector backbones were reaction cleaned-up and restricted with *Nhe*I (NEB, # R0131L) and *Sac*I (NEB, #R0156L) for 8h at 37°C. Ten units of *Dpn*I (NEB, #R0176) and 1 unit of Shrimp Alkaline Phosphatase (NEB, #M0371) was added to the same reaction and incubated for 60min at 37°C. The vector backbone was then purified from 1% agarose gel. In two separate reactions, the insert (586bp *vif*) and vectors (5.914kb pSA-6His or 5.853kb pSA-6His-RIL) were ligated, transformed into chemically competent DH5α *E*. *coli* cells, and selected on ampicillin-containing LB-agar plates after 18h incubation at 30°C. Ten randomly selected bacterial colonies were subjected to colony PCR using vector-specific pMXB10-up101-F and insert-specific Vif-R primers (**Table 3**). Transformants that contained the amplicons of expected size by PCR were then verified using DNA restriction and sequence analyses.

### Construction of pSA-C6His-RIL

Vector pSA-C6His-RIL was constructed by replacing *nef* gene with a multiple cloning site (MCS). The MCS introducing *Bgl*II, *Nhe*I, *Eco*RI, *Nru*I, *Sac*II, *Sal*I, and *Not*I restriction sites was constructed by annealing two oligonucleotides, MCS-F and MCS-R (**Table 3**). The oligonucleotides anneal to create *Nde*I and *Sac*I cohesive ends for ligation into a pSA-6His-RIL vector restricted with 5′ *Nde*I and 3′ *Sac*I sites, maintaining the correct frame for the C-terminal 6His tag and stop codon. The MCS-F and MCS-R oligonucleotides were resuspended in annealing buffer (10mM Tris, pH 7.5–8.0, 50mM NaCl, 1 mM EDTA) and mixed in equimolar concentrations (200μM each) in 50μl reaction. The annealing reaction was incubated for 5 min in a hot block at 90°C. The block was removed from the heat source and the reaction was left to gradually cool to room temperature for 45 min and stored at -20°C. The pSA-HNef-6His-RIL vector (5μg) was restricted with *Nde*I/ *Sac*I at 37°C for 4h and 5.837kb vector backbone (pSA-6His-RIL) was purified from 1% agarose gel. Annealed oligonucleotides were diluted 1:10 with nuclease-free water and ligated with *Nde*I/ *Sac*I-restricted vector in a 4:3 molar ratio. Ligation reaction was transformed into DH5α *E*. *coli* cells, and selected on ampicillin-containing LB-agar plates after 18h incubation at 30°C. Randomly selected bacterial colonies were used to prepare plasmid minipreps, which were subjected to DNA restriction and sequence analyses.

### Expression of Nef/P24/Vif in pSA-HNef/P24/Vif-6His or pSA-HNef/P24/Vif-6His-RIL-transformed NiCo21(DE3) *E*. *coli*

Sequencing-confirmed pSA-HNef/P24/Vif-6His or pSA-HNef/P24/Vif-6His-RIL was transformed into chemically competent NiCo21(DE3) *E coli* and transformants selected on ampicillin-containing LB-Agar plates. For expression experiments, a single colony from a freshly streaked (18–22h) plate was inoculated into 10 ml of ampicillin-supplemented LB broth. The starter culture was grown at 30°C while shaking at 250rpm until OD_600_ reached to ~1.0. The cultures were centrifuged at 3000xg for 10 min, re-suspend into fresh ampicillin-containing LB broth, and used to inoculate the main culture at a 1:20 dilution (~0.5 OD_600_) in a baffled flask. The culture was grown at 30°C while shaking at 250 rpm until OD_600_ reached to 0.5–0.6. The cultures were then equilibrated to induction temperature and expression was induced with various concentrations of Isopropylthio-β-galactoside (IPTG). Induced cultures were grown for different time lengths (6h at 30°C; 12h at 22°C; 16h at 18°C) before pelleting bacterial cells by centrifugation at 5000xg for 10 min in pre-weighed centrifuge tubes/ bottles.

### Expression of proteins in NiCo21(DE3) transformed with pACYC-RIL, pRARE2, and pLysSRARE2

NiCo21(DE3) bacteria were individually transformed with pACYC-RIL, pRARE2, and pLysSRARE2 vectors and the transformants selected on chloramphenicol-containing LB agar plates. Transformants were grown and competent cells were prepared according to the method of Inoue [20]. The pACYC-RIL, pRARE2, and pLysSRARE2-containing NiCo21(DE3) were then transformed with pSA-HNef/P24/Vif-6His vectors and the transformants selected on LB agar plates containing both ampicillin and chloramphenicol. Cultures were expanded and the expression protocol given above was essentially followed.

### Bacterial cell lysis and protein extraction

To every gram of bacterial cell pellet, 4ml of B-PER extraction reagent (Thermo Scientific, #78248) supplemented with DNAse I (Thermo Scientific, #90083) and protease cocktail (Thermo Scientific, #87785) was added. The suspension was incubated at 22°C for 60min with gentle shaking. Soluble and insoluble proteins were partitioned by centrifuging bacterial cell lysate at 15,000xg for 10 min at 4°C. Clear supernatant containing soluble proteins was passed through a 0.45μm membrane (Millipore, #HPWP04700) and used for immobilized-metal affinity chromatography (IMAC). Insoluble lysed bacterial biomass was resuspended to its original volume with Tris-buffered saline (TBS). Both, soluble and insoluble fractions were stored in small aliquots at -80°C for further analysis.

### Protein assays, SDS-PAGE, and immunoblotting

Total protein was quantified using the Qubit Protein Assay Kit (Life Technologies – Invitrogen, # Q33211) in a Qubit fluorometer (Life Technologies – Invitrogen). For SDS-PAGE separation, protein fractions were mixed with 5x reducing sample buffer (Thermo Scientific, #39000), resolved on 12% (w/v) polyacrylamide gels, and visualized by staining with Coomassie blue G250 [21]. For immunoblot analysis, proteins were electroblotted onto Hybond ECL nitrocellulose membrane (GE Healthcare Life Sciences, #RPN2020D). The membrane was washed in Tris-buffered saline (TBS) for 5 min, blocked with 5% nonfat milk in TTBS (TBS with 0.1% Tween 20) for 1h by shaking at room temperature, and probed with either primary anti-Nef MAb (Thermo Scientific, #MA1-71507) or anti-His MAb (Thermo Scientific, #MA1-213157) with shaking at 4°C overnight. After washing with TTBS, membrane was incubated with secondary HRP-conjugated IgG (H+L) antibody (Thermo Scientific, #32430) and protein bands were detected using SuperSignal West Pico Chemiluminescent substrate (Thermo Scientific, #34080). Images were captured using FluorChem M Imager (Protein Simple).

### Purification of recombinant proteins

Recombinant HIV-1 Nef protein was purified in two steps. First, the soluble protein fraction was pre-adsorbed onto chitin resin (NEB, #S6651) to capture bacterial Histidine-rich proteins. An appropriate amount of Chitin beads was added into Econo-Pac Chromatography Column (BioRad, #732–1010) and equilibrated in two resin-bed volumes of Equilibration/Wash Buffer (50mM sodium phosphate, 500mM sodium chloride and 10mM imidazole, pH 7.4). Soluble protein fraction was mixed with an equal volume of Equilibration/ Wash Buffer and added to the equilibrated chitin beads (1 ml of chitin resin for each volume of lysate corresponding to 1 gram of NiCo21(DE3) cell pellet). The column was then placed on an end-over-end rotator and revolved for 30min at 4°C. Void volume containing target protein was eluted by gravity flow and used for IMAC purification step.

For IMAC, HisPur Cobalt resin (Thermo Scientific, #89965) was used following the manufacturer’s protocol. Briefly, an appropriate amount of cobalt resin was added in a 15ml centrifuge tube and washed with two resin-bed volumes of Equilibration/ Wash Buffer. The chitin bead pre-adsorbed soluble protein fraction was combined with equilibrated cobalt resin and mixed on an end-over-end rotator for 60min at 4°C. The resin was then washed with Equilibration/Wash Buffer until the absorbance at 280nm reached to the baseline. Bound protein was eluted using one resin-bed volume of Elution Buffer (50mM sodium phosphate, 500mM sodium chloride, 150mM imidazole, pH 7.4). This step was repeated 2–3 times while saving individual fractions. Fractions were analyzed by SDS-PAGE/Western blot, dialyzed against phosphate buffered saline (PBS) using Slide-A-Lyzer Dialysis Cassettes, 7K MWCO (Thermo Scientific, #66710) and stored at -80°C in small aliquots.

## Results

### Construction of a plasmid expressing HIV-1 Nef

**Fig 1:**
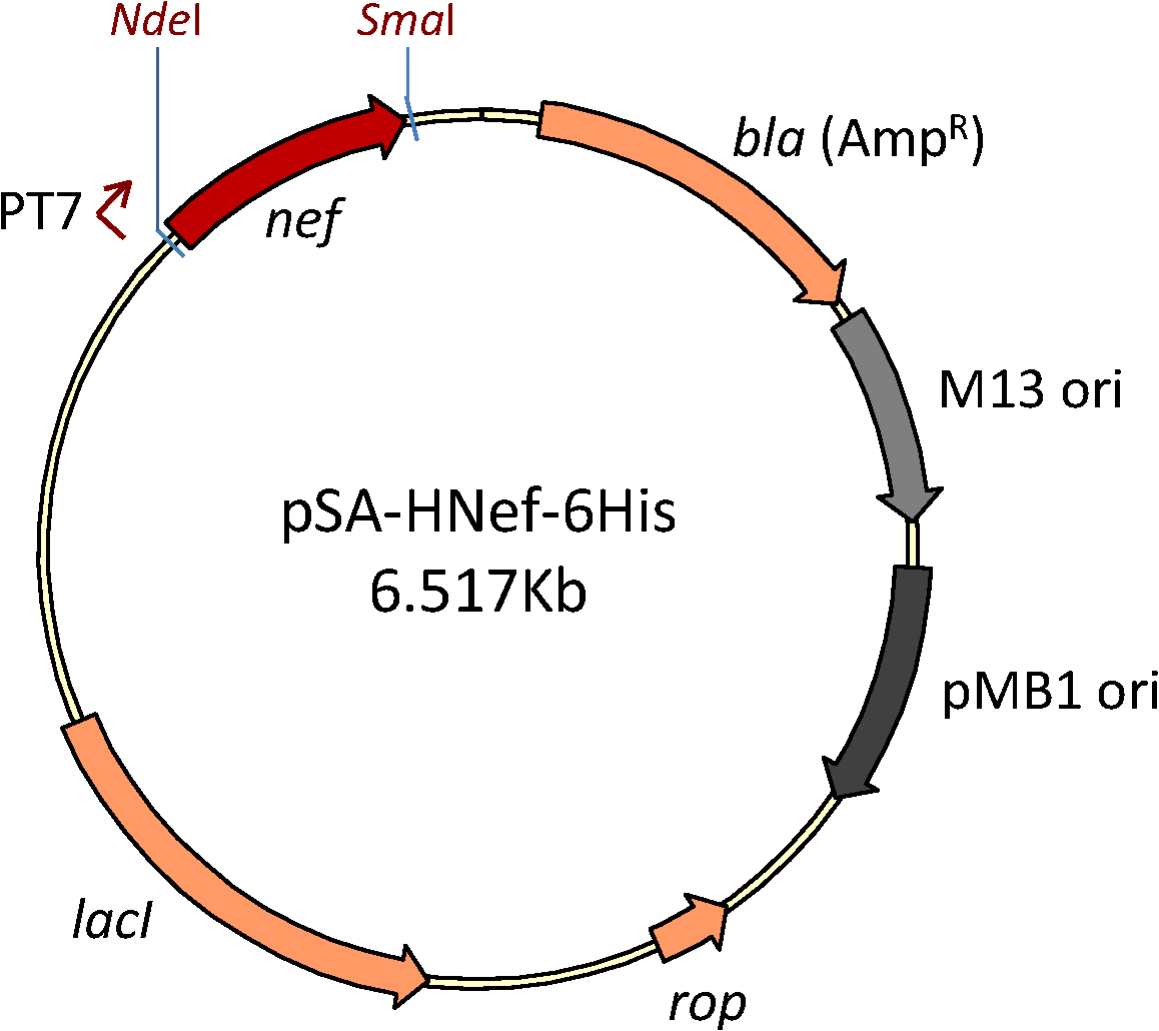
Schematic representation of expression vector pSA-HNef-6His. The *nef* gene was PCR amplified from pNL4.3 plasmid, restricted with *Nde*I/ *Sac*I, and ligated into pSA-6His vector at the same sites.

pSA-HNef-6His vector (**Fig 1**) used to produce HIV-1 Nef protein was constructed by modifying the pSA-Hp24-6His vector [13]. The HIV-1 *p*24 coding sequence was removed by restricting the plasmid with *Nde*I and *Sac*I, and replaced with HIV-1 *nef* coding sequence at the same sites. The resulting 6.517kb vector was verified by DNA restriction and sequence analyses (data not shown). The mRNA sequence encoding the *nef* gene is transcribed from an inducible T7 promoter and the resulting protein has a C-terminal 6His tag for IMAC-mediated purification.

### Expression of Nef in pSA-HNef-6His-transformed NiCo21(DE3) *E*. *coli*

**Fig 2:**
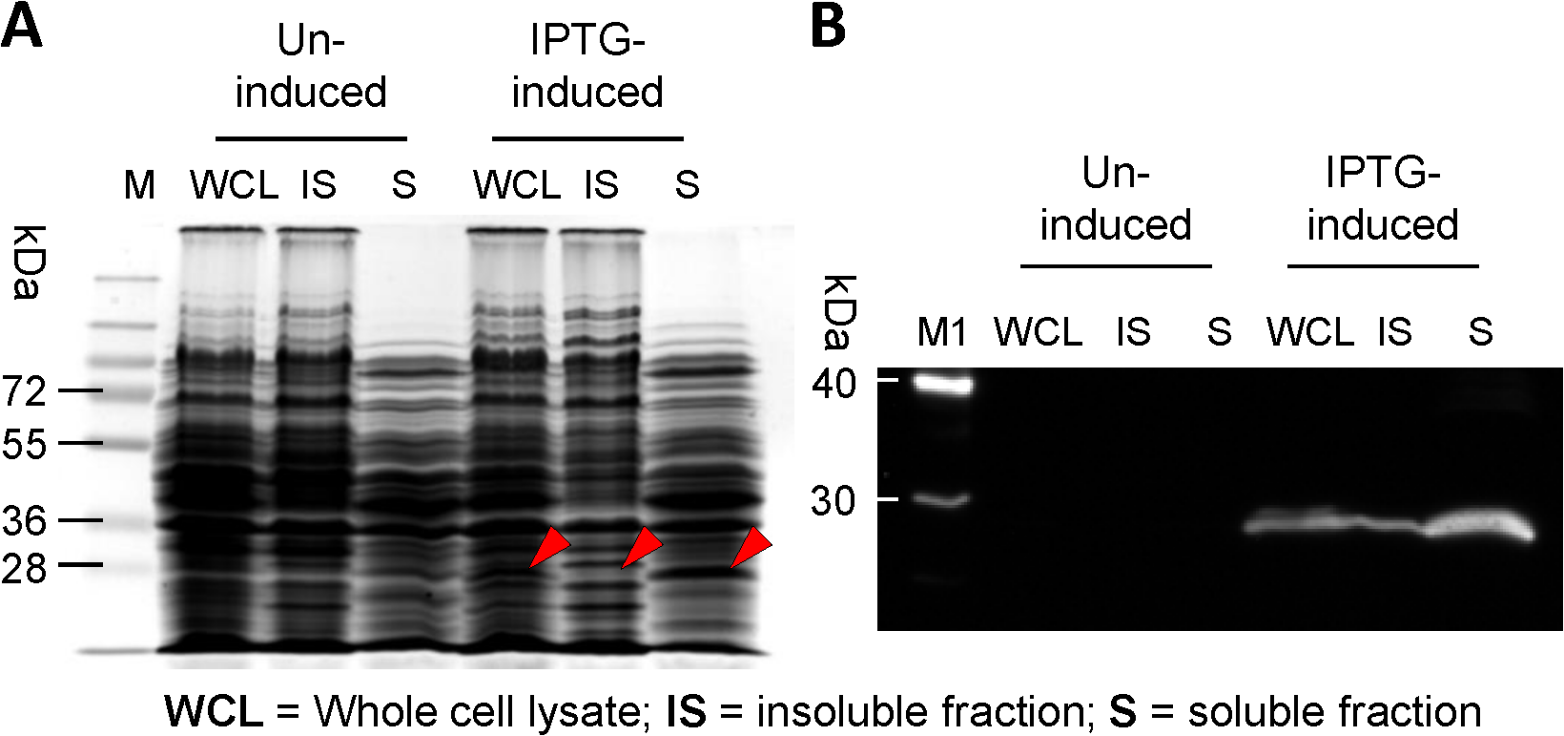
SDS-PAGE and immunoblot analysis of recombinant Nef. NiCo21(DE3) *E*. *coli* were transformed with pSA-HNef-6His vector and grown overnight from a single colony at 30°C in LB broth supplemented with 100 μg/ml ampicillin. The cultures were diluted 100-fold in the same medium and grown to mid-log phase (OD_600_ ~0.5–0.6), at which point IPTG was added to a final concentration of 0.4 mM. The induced cells were grown for another 6 h at 30°C and stored on ice. Nine microliters of samples were mixed with 4 x loading dye, electrophoretically resolved on a 12% SDS-PAGE gel and analyzed by (**A**) Coomassie staining and (**B**) immuno-blotting. Lanes: M, PageRuler Prestained Protein Ladder Plus; WCL, whole cell lysate; IS, insoluble fraction; S, soluble fraction; M1, MagicMark XP Western Protein Standard. Recombinant Nef was produced in the induced cells but not in the un-induced controls, and mainly present in the soluble fraction.

*E*. *coli* NiCo21(DE3) was used to express 6His-tagged HIV-1 Nef using shaker flask culture conditions for expression. Plasmid-borne retroviral sequences are unstable in *E*. *coli* when grown at 37°C [22]. Therefore, the cultures of pSA-HNef-6His-transformed NiCo21(DE3) were grown at 30°C until the OD_600_ reached 0.5–0.6. Cultures were then induced with 0.4mM IPTG and incubated for another 6h at 30°C. When subjected to SDS-PAGE analysis, the expressed Nef protein appeared as an approximately 27kDa band in the IPTG-induced fractions (**Fig 2A**). Overexpressed Nef protein was mainly present in the soluble fraction. However, immunoblot analysis with the anti-Nef antibody revealed that some Nef was also present in the insoluble fraction (**Fig 2B**).

### Optimization of IPTG concentration and induction temperature

**Fig 3:**
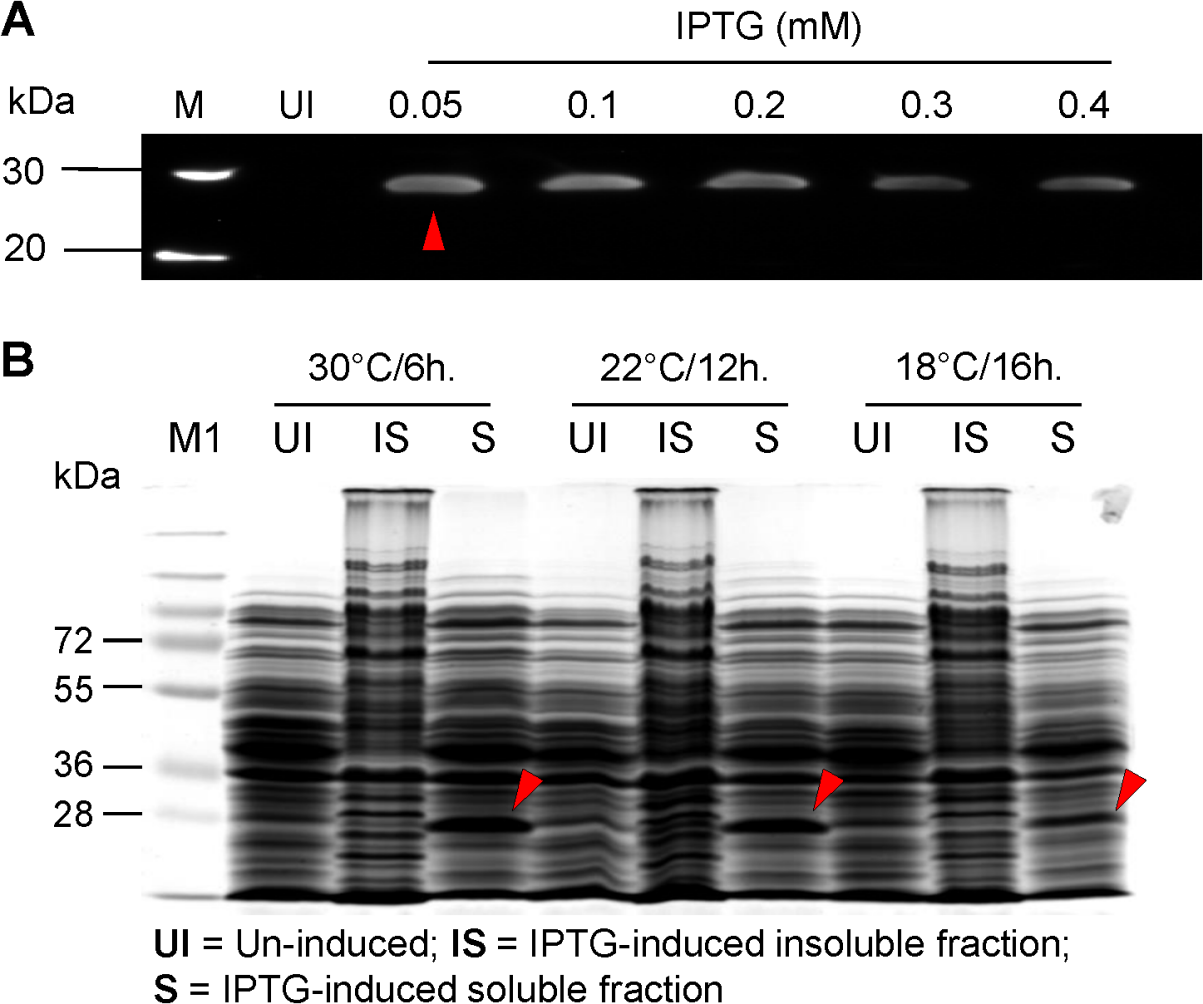
Optimization of IPTG concentration and induction temperature. (**A**) For optimization of IPTG concentration, the overnight cultures of pSA-HNef-6His-transformed NiCo21(DE3) were diluted 1:100 in LB+Amp (100 μg/ml) and grown to mid-log phase (OD_600_ ~0.5–0.6). The cultures were then induced with varying concentrations (0, 0.05, 0.1, 0.2, 0.3, and 0.4 mM) of IPTG and grown for another 6 h at 30°C. Nine microliters of samples were mixed with 4x loading dye, electrophoretically resolved on a 12% SDS-PAGE gel and analyzed by immuno-blotting. A concentration of 0.05 mM IPTG (red arrow) was sufficient to induce high level Nef expression. (**B**) For optimal induction temperature, the overnight cultures of pSA-HNef-6His-transformed NiCo21(DE3) were diluted 1:100 in LB+Amp (100 μg/ml) and grown to mid-log phase (OD_600_ ~0.5–0.6). The cultures were then induced with 0.05 mM of IPTG and grown at 30, 22, and 18°C for 6, 12, and 16 h respectively. Nine microliters of samples were mixed with 4 x loading dye, electrophoretically resolved on a 12% SDS-PAGE gel and analyzed by Coomassie staining. Nef expression was similar at 30 and 22°C but lower at 18°C. Lanes: M, BenchMark Pre-stained protein ladder; M1, PageRuler Prestained Protein Ladder Plus; 1, un-induced NiCo21 at 30°C (soluble); 2, IPTG-induced NiCo21 at 30°C (insoluble); 3, IPTG-induced NICo21 at 30°C (soluble); 4, un-induced NiCo21 at 22°C (soluble); 5, IPTG-induced NiCo21 at 22°C (insoluble); 6, IPTG-induced NiCo21 at 22°C (soluble); 7, un-induced NiCo21 at 18°C (soluble); 8, IPTG-induced NiCo21 at 18°C (insoluble); 9, IPTG-induced NiCo21 at 18°C (soluble).

In order to optimize Nef expression, we tested ranges of IPTG concentrations (0–0.4mM) and incubation temperatures (30, 22, and 18°C). Expression of HIV-1 Nef was optimal at the lowest tested concentration of IPTG *i*.*e*. 0.05mM as determined by the western blot analysis (**Fig 3A**). HIV-1 Nef production was similar in 0.05mM IPTG-induced cultures when grown at 30°C and 22°C, but lower in 18°C-grown cultures (**Fig 3B**). However, neither IPTG concentration nor various incubation temperatures enhanced overall Nef production in *E*. *coli* NiCo21(DE3) cells.

### Effect of rare tRNA supplementation on Nef expression in NiCo21(DE3) *E*. *coli*

**Fig 4:**
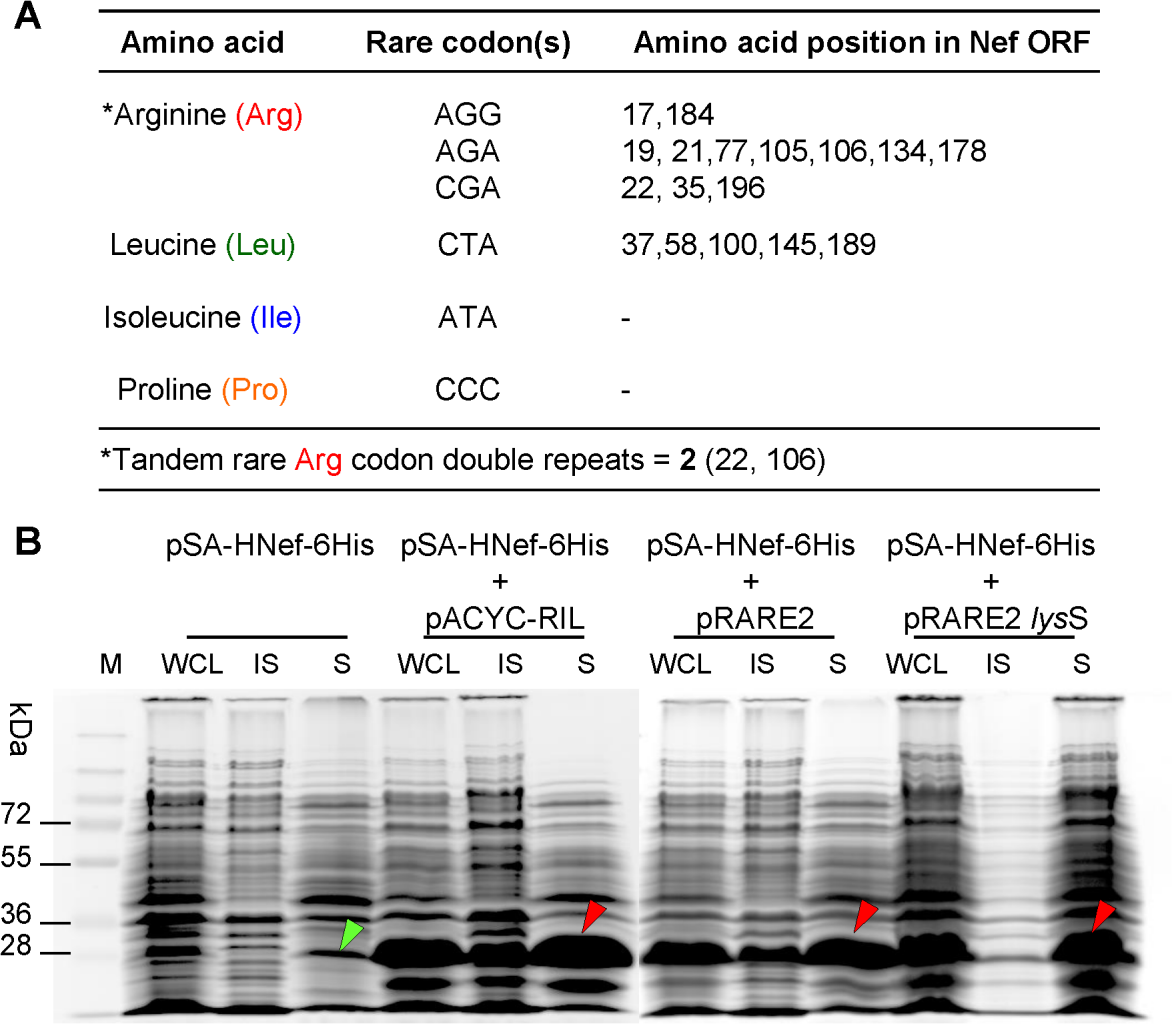
Effect of rare tRNA supplementation on Nef expression in NiCo21(DE3) *E*. *coli*. (**A**) ORF coding for HIV-1 *nef* gene was subjected to rare codon analysis using an online tool ‘*Rare Codon Calculator (RaCC)*’ http://nihserver.mbi.ucla.edu/RACC/. Three rare codons encoding arginine and one rare codon encoding leucine are present at 17 amino acid positions in Nef ORF. (**B**) NiCo21(DE3) *E*. *coli* were individually transformed with rare tRNA-expressing helper plasmids (pACYC-RIL, pRARE2, pRARE2-lysS) and selected on LB+Cam. These bacteria were then made competent, transformed with pSA-HNef-6His, and selected on LB+Cam+Amp plates. Cultures were grown overnight from a single colony at 30°C in LB+Cam+Amp. The cultures were then diluted 100-fold in the same medium and grown to mid-log phase (OD_600_ ~0.5–0.6), at which point IPTG was added to a final concentration of 0.05 mM. The induced cells were grown for another 12 h at 22°C and stored on ice. Nine microliters of samples were mixed with 4 x loading dye, electrophoretically resolved on a 12% SDS-PAGE gel analyzed by Coomassie staining. Expression of rare codon tRNA genes resulted in high level expression of Nef. Lanes: M, PageRuler Prestained Protein Ladder Plus; WCL, whole cell lysate; IS, insoluble fraction; S, soluble fraction.

Over-expression of heterologous proteins in *E*. *coli* may be considerably circumscribed by the presence of "rare" codons in the foreign mRNA that are seldom used by *E*. *coli* [23, 24]. When subjected to ‘rare’ codon analysis, the 618bp coding sequence for HIV-1 Nef was found to contain **(a)** three rare codons (AGG, AGA, CGA) for arginine at positions 17, 19, 21, 22, 35, 77, 105, 106, 134, 178, 184, 194, and **(b)** one rare codon (CTA) for leucine at positions 37, 58, 100, 145, and 189 (**Fig 4A**). To investigate whether Nef expression could be improved by expressing *nef* together with rare tRNA genes, NiCo21(DE3) were transformed with rare tRNA expressing pACYC-RIL, pRARE2, and pLysSRARE2 helper plasmids. The pACYC-RIL plasmid supplies tRNA for four rare codons (AUA, AGG, AGA, CUA), whereas pRARE2 supplies those for seven rare codons (AUA, AGG, AGA, CUA, CCC, CGG, and GGA). In addition to seven rare codons, transformation with pLysSRARE2 also results in lower background expression due to the expression of T7 lysozyme. When expressed in the presence of rare tRNA supplying helper plasmids, Nef expression strikingly improved as shown in **Fig 4B**. However, this also lead some Nef protein to end up in the insoluble fractions, suggesting that bacterial protein folding machinery was saturated by the enhanced expression. There was no significant change in Nef expression between the NiCo21(DE3) containing pACYC-RIL and pRARE2/ pLysSRARE2, suggesting that supplementation with four rare codons was sufficient to optimize Nef expression. In a side-by-side comparison, NiCo21(DE3)/pACYC-RIL showed relatively higher production of soluble Nef (**Fig 4B**).

In attempts to shift the relative soluble/insoluble protein production, protein expression was tested at 18°C, but it did not alter the distribution of recombinant protein between soluble and insoluble fractions, nor did the production of soluble Nef improve further (data not shown). Another modification was tested by the addition of 2–3% ethanol to the culture medium [25, 26] and incubation at 42°C [27]. These conditions lead to overexpression of bacterial chaperones, which may result in improved folding and enhanced solubility of overexpressed recombinant proteins. However these approaches failed to generate more soluble Nef with our production system (data not shown). Expression of Nef protein in the presence or absence of rare tRNA genes had minimal effect on the bacterial growth as determined by the total biomass yield (~12 g/L and 12.5 g/L for NiCo21(DE3) transformed with pSA-HNef-6His, and pSA-HNef-6His + pACYC-RIL respectively, when grown in LB broth.

### Construction of pSA-HNef-6His-RIL for concomitant expression of Nef and rare tRNA genes

**Fig 5:**
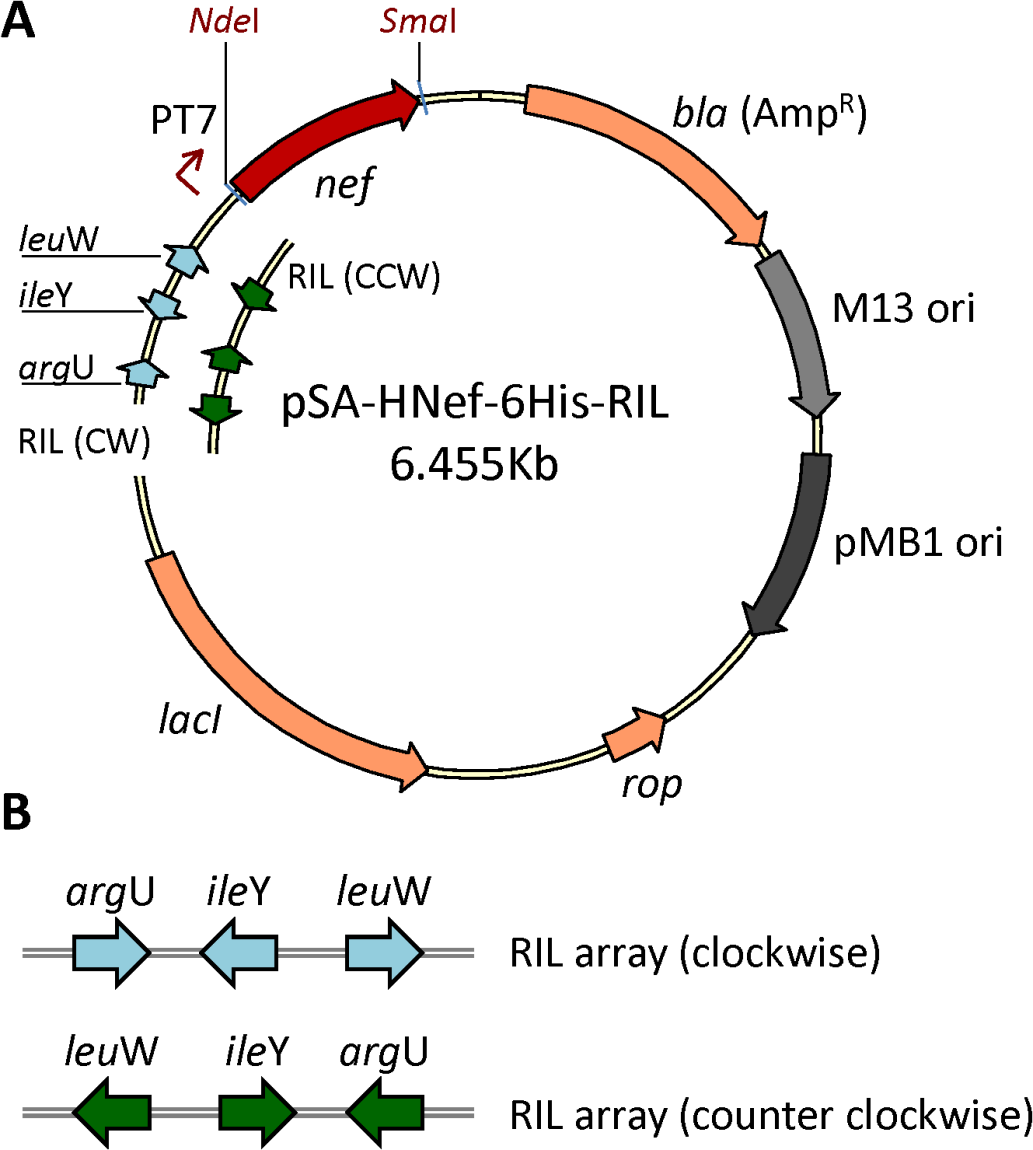
Schematic representation of expression vector pSA-HNef-6His-RIL. **A.** Map of pSA-HNef-6His-RIL vector with rare tRNA genes array. **B.** Arrangement of rare tRNA genes in clockwise and counter clockwise orientation.

In order to alleviate issues associated with coexistence of tRNA helper plasmids with expression plasmids, we modified pSA-HNef-6His to express rare tRNA genes in concomitant with *nef* gene. The modified pSA-HNef-6His-RIL plasmid was constructed by exchanging 936bp non-essential DNA fragment between the *lac*I gene and T7 promoter with 874bp DNA fragment containing *arg*U, *ile*Y, and *leu*W rare tRNA genes. Blunt-end ligation of rare tRNA gene fragment into the vector resulted in a plasmid with tRNA gene array arranged in either clockwise (CW) or counter-clockwise (CCW) orientation relative to the T7 promoter (**Fig 5A and 5B**).

### Expression of Nef in NiCo21(DE3) transformed with pSA-HNef-6His-RIL

**Fig 6:**
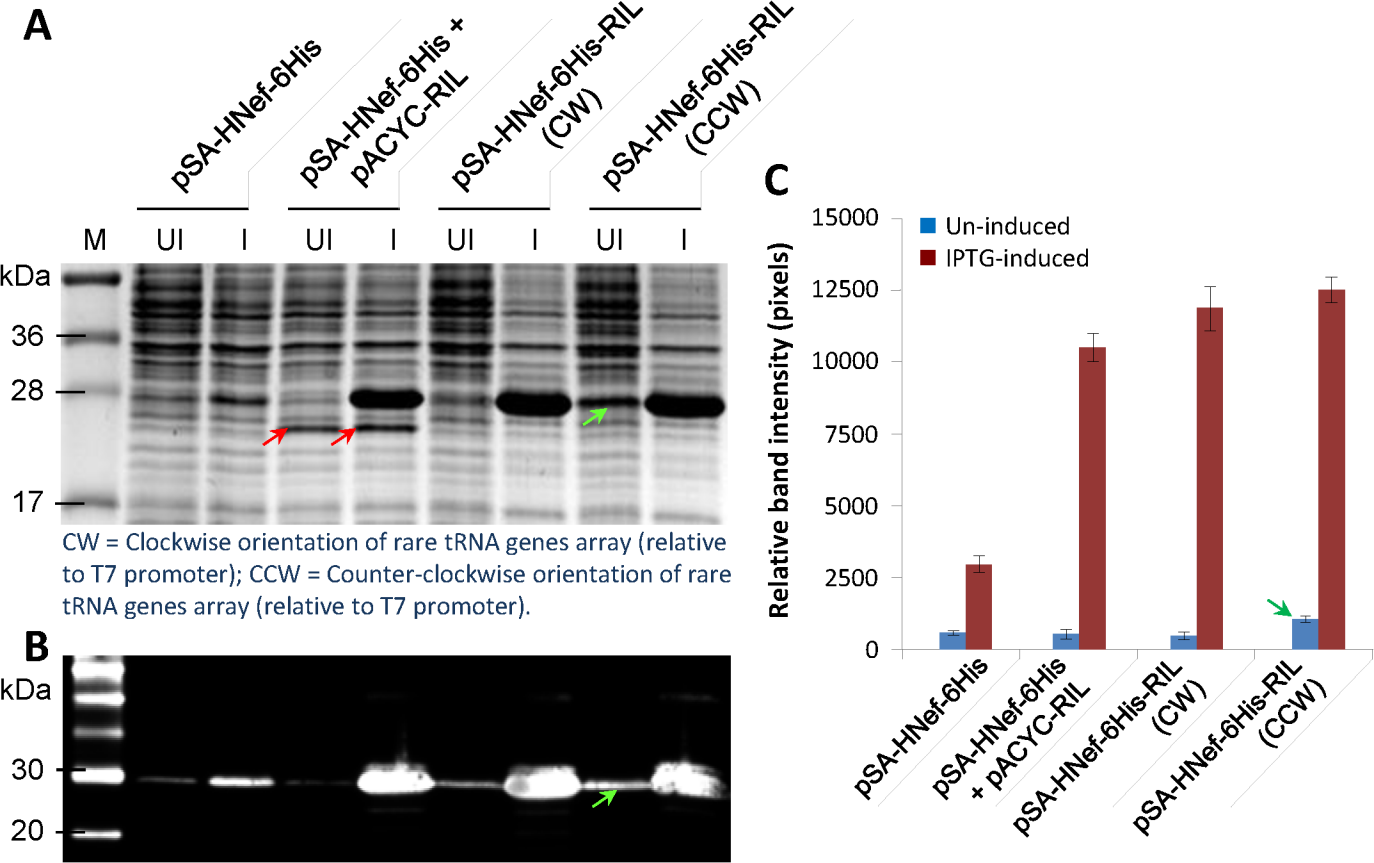
Expression of Nef from pSA-HNef-6His vectors with or without rare tRNA genes array. To evaluate the effect of the introduction of rare tRNA genes array in pSA-HNef-6His vector, the NiCo21 *E*. *coli* were individually transformed with pSA-HNef-6His, pSA-HNef-6His-RIL(CW), and pSA-HNef-6His-RIL(CCW) and Nef expression was compared with NiCo21 *E*. *coli* co*-*transformed with pSA-HNef-6His and rare tRNA expressing pACYC-RIL vectors. The cultures were grown overnight at 30°C in LB broth containing Amp (for bacteria harboring pSA-HNef-6His or pSA-HNef-6His-RIL) or Amp+Cam (for bacteria harboring pSA-HNef-6His + pACYC-RIL). The cultures were then diluted 100-fold in the same medium and grown to mid-log phase (OD_600_ ~0.5–0.6), at which point IPTG was added to a final concentration of 0.05 mM. The induced cells were grown for another 12 h at 22°C and stored on ice. Nine microliters of samples were mixed with 4 x loading dye, electrophoretically resolved on a 15% SDS-PAGE gel and analyzed by (**A**) Coomassie staining, (**B**) immuno-blotting and (**C**) band densitometry. Nef expression in NiCo21 transformed with pSA-HNef-6His-RIL (CW/CCW) was comparable with NiCo21 co-transformed with pSA-HNef-6His and pACYC-RIL. A prominent band running at 25 kDa (red arrows) appears in NiCo21 harboring pSA-HNef-6His/ pACYC-RIL. This is most probably chloramphenicol acetyltransferase monomer. Higher baseline expression for un-induced cultures were noted in NiCo21 harboring pSA-HNef-6His-RIL(CCW) (see green arrows). This suggests that ileY tRNA gene most probably doesn’t contain a transcription terminator downstream and the baseline expression of Nef is a result of transcriptional read-through of mRNA synthesis from *ile*Y promoter.

We expressed Nef in *E*. *coli* transformed with pSA-HNef-6His-RIL(CW)/(CCW) and compared Nef production with *E*. *coli* transformed with pSA-HNef-6His (served as negative control), and pSA-HNef-6His together with pACYC-RIL (served as positive control). Expression experiments were carried out using shaker flask culture conditions for expression as described above. When subjected to SDS-PAGE analysis, Nef expression in bacteria containing pSA-HNef-6His-RIL was comparable with those that contained both pSA-HNef-6His and pACYC-RIL. Little Nef produced in bacteria that harbored pSA-HNef-6His vector (**Fig 6A**). There was noticeable background expression in un-induced cultures from pSA-HNef-6His-RIL (CW) compared to pSA-HNef-6His-RIL (CCW) (**green arrow, Fig 6A and 6B**). This suggests that in addition to T7 promoter, Nef was also expressed under the control of some promoter upstream of T7 promoter, most likely of *ile*Y (**Fig 5B**). As a result, the pSA-HNef-6His-RIL(CCW) was not used in subsequent expression experiments. Samples from pSA-HNef-6His+pACYC-RIL showed a prominent 25kDa band present in both un-induced, and IPTG-induced cultures (**red arrows, Fig 6A**). This band most probably represents chloramphenicol acetyltransferase monomer expressed from pACYC-RIL vector. Proteins were also subjected to immunoblot analysis (**Fig 6B**) using anti-Nef MAb as primary antibody and relative band intensities were determined and plotted as shown in **Fig 6C**. Together, these experiments show that recombinant protein can be produced from a single vector concomitantly expressing rare tRNA and a heterologous gene, and in amounts comparable (or better) to traditional two-vector system.

### Construction of pSA-HP24-6His-RIL for concomitant expression of P24/CA and rare tRNA genes

**Fig 7:**
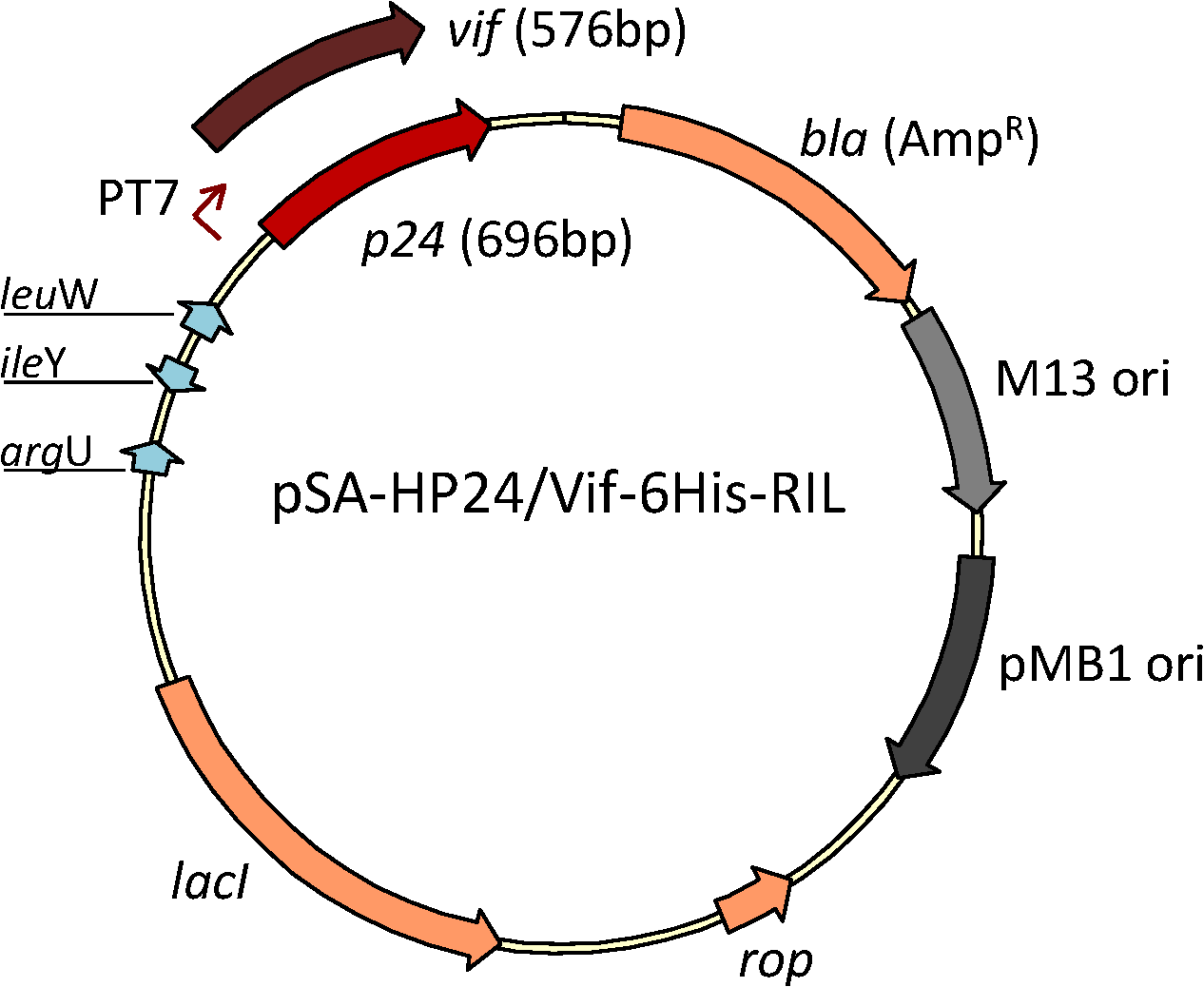
Schematic representation of expression vectors pSA-HP24/Vif-6His-RIL. Map shows the vector containing HIV-1 *p24* or *vif* genes cloned downstream T7 promoter.

**Fig 8:**
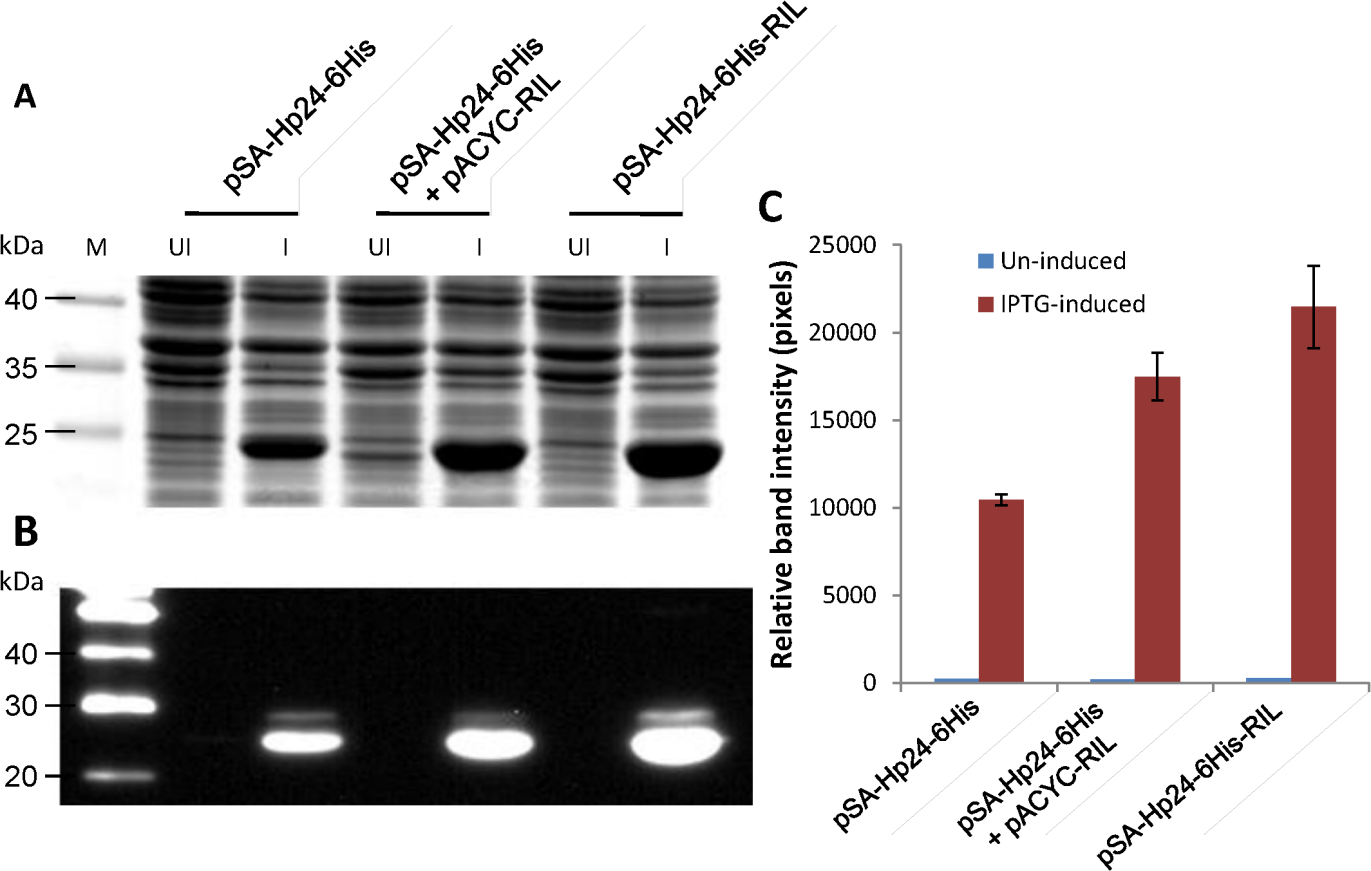
Expression of HIV-1 P24 from pSA-HP24-6His vectors with or without rare tRNA genes array. To evaluate the effect of the introduction of rare tRNA genes array in pSA-HP24-6His vector, the NiCo21(DE3) *E*. *coli* were individually transformed with pSA-HP24-6His, and pSA-HP24-6His-RIL and P24 expression was compared with NiCo21(DE3) *E*.*coli* co*-*transformed with pSA-Hp24-6His and rare tRNA expressing pACYC-RIL vectors. The cultures were grown overnight at 30°C in LB broth containing Amp (for bacteria harboring pSA-HP24-6His or pSA-HP24-6His-RIL) or Amp+Cam (for bacteria harboring pSA-HP24-6His + pACYC-RIL). The cultures were then diluted 100-fold in the same medium and grown to mid-log phase (A600 ~0.5–0.6), at which point IPTG was added to a final concentration of 0.05mM. The induced cells were grown for another 12 hours at 22°C and stored on ice. Three microliters of samples were mixed with 4X loading dye, electrophoretically resolved on a 12% SDS-PAGE gel and analyzed by Coomassie staining **(A)**, immuno-blotting **(B)**, and band densitometery **(C)**. The P24 expression in NiCo21 transformed with pSA-HP24-6His-RIL was comparable with NiCo21 co-transformed with pSA-HP24-6His and pACYC-RIL.

In order to further confirm the hypothesis that recombinant protein(s) can be produced in similar or better amounts from a single vector concomitantly expressing rare tRNA and a heterologous gene compared to traditional two-vector system, we expressed HIV-1 *p24* gene. HIV-1 *p24* gene contains **(a)** three rare codons (AGG, AGA, CGA) for arginine at positions 44, 59, 62, 94, 105, 116, 124, 129, 135, 191; **(b)** one rare codon (CTA) for leucine at positions 18, 134, 173; **(c)** one rare codon (ATA) for isoleucine at positions 66, 77, 96, 103, 115; and **(d)** one rare codon (CCC) for proline at positions 122, 186. We have previously shown that pACYC-RIL plasmid significantly helped with improving the overall yields of P24/CA [13]. Here we replaced the *nef* gene in pSA-HNef-6His-RIL with *p24* and compared the expression profile of P24/CA from pSA-HP24-6His, pSA-HP24-6His together with pACYC-RIL, and pSA-HP24-6His-RIL (**Fig 7**). The helper plasmids pRARE2/pRARE2 *lys*S were omitted because in the previous work they were found not to offer any advantage over pACYC-RIL helper plasmid [13]. As anticipated, almost 2 fold more P24/CA was expressed in cultures containing pSA-HP24-6His+pACYC-RIL and pSA-HP24-6His-RIL. The SDS-PAGE (**Fig 8A**), Western Blot (**Fig 8B**), and densitometric (**Fig 8C**) analyses showed that relatively more p24/CA was produced from the single plasmid (pSA-HP24-6His-RIL) compared to traditional two plasmid (pSA-HP24-6His+pACYC-RIL) system.

### Construction of pSA-HVif-6His-RIL for concomitant expression of Vif and rare tRNA genes

**Fig 9:**
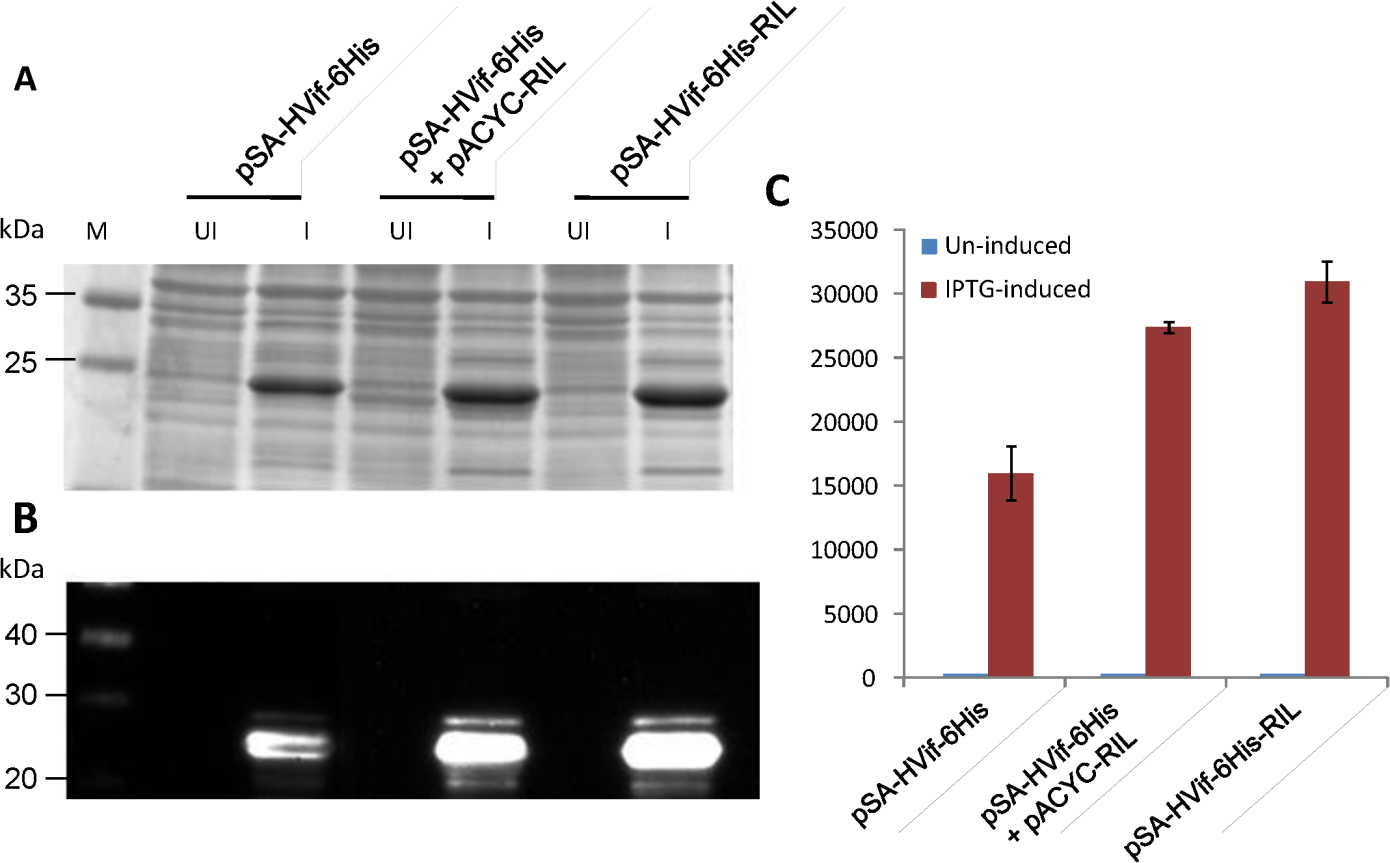
Expression of HIV-1 Vif from pSA-HVif-6His vectors with or without rare tRNA genes array. To evaluate the effect of the introduction of rare tRNA genes array in pSA-HVif-6His vector, the NiCo21(DE3) *E*. *coli* were individually transformed with pSA-HVif-6His, and pSA-HVif-6His-RIL and Vif expression was compared with NiCo21(DE3) *E*.*coli* co*-*transformed with pSA-HVif-6His and rare tRNA expressing pACYC-RIL vectors. The cultures were grown overnight at 30°C in LB broth containing Amp (for bacteria harboring pSA-HVif-6His or pSA-HVif-6His-RIL) or Amp+Cam (for bacteria harboring pSA-HVif-6His + pACYC-RIL). The cultures were then diluted 100-fold in the same medium and grown to mid-log phase (A600 ~0.5–0.6), at which point IPTG was added to a final concentration of 0.05mM. The induced cells were grown for another 12 hours at 22°C and stored on ice. Three microliters of samples were mixed with 4X loading dye, electrophoretically resolved on a 12% SDS-PAGE gel and analyzed by Coomassie staining **(A)**, immuno-blotting **(B)**, and band densitometery **(C)**. The Vif expression in NiCo21(DE3) transformed with pSA-HVif-6His-RIL was comparable with NiCo21 co-transformed with pSA-HVif-6His and pACYC-RIL.

To further demonstrate the utility of the single plasmid concurrently expressing gene of interest and rare tRNA genes, we exchanged *nef* gene with HIV-1 *vif* gene in pSA-HNef-6His and pSA-HNef-6His-RIL vectors. The *vif* gene contains **(a)** two rare codons (AGG, AGA) for arginine at positions 6, 17, 19, 25, 35, 43, 79, 92, 95, 123, 134, 169, 175, 186; **(b)** one rare codon (CTA) for leucine at positions 61 104 108 147 152; **(c)** one rare codon (ATA) for isoleucine at positions 53 68 89 122 126 130 156 161; and **(d)** one rare codon (CCC) for proline at positions 179. Expression of Vif was compared between the cultures that contained pSA-HVif-6His, pSA-HVif-6His together with pACYC-RIL, and pSA-HVif-6His-RIL (**Fig 7**). As shown in **Fig 9A, 9B and 9C**, around two fold more Vif was produced in cultures containing pSA-HVif-6His+pACYC-RIL and pSA-HVif-6His-RIL compared to pSA-HVif-6His. Consistent with previous observations for Nef and P24, relatively more Vif was produced from the vector that expresses both the heterologous gene and rare tRNA genes. However, unlike Nef and P24, Vif expressed as insoluble protein and optimizing the IPTG concentration and temperature did not yield any soluble Vif (data not shown).

### Growth profile, expression, and purification of HIV-1 Nef and P24 in NiCo21(DE3) transformed with pSA-HNef-6His-RIL and pSA-HVif-6His RIL

By expressing three rare codon –contain HIV-1 genes i.e. *nef*, *p24*, and *vif*, we showed the suitability of our proposed vector that concomitantly expresses the gene of interest and rare tRNA genes. Here we compared the growth profile, expression, and purification HIV-1 Nef and P24 between traditional two-plasmid system with that of single expression plasmid system. Because HIV-1 Vif failed to express as soluble protein, we have omitted it from this set of experiments.

**Fig 10:**
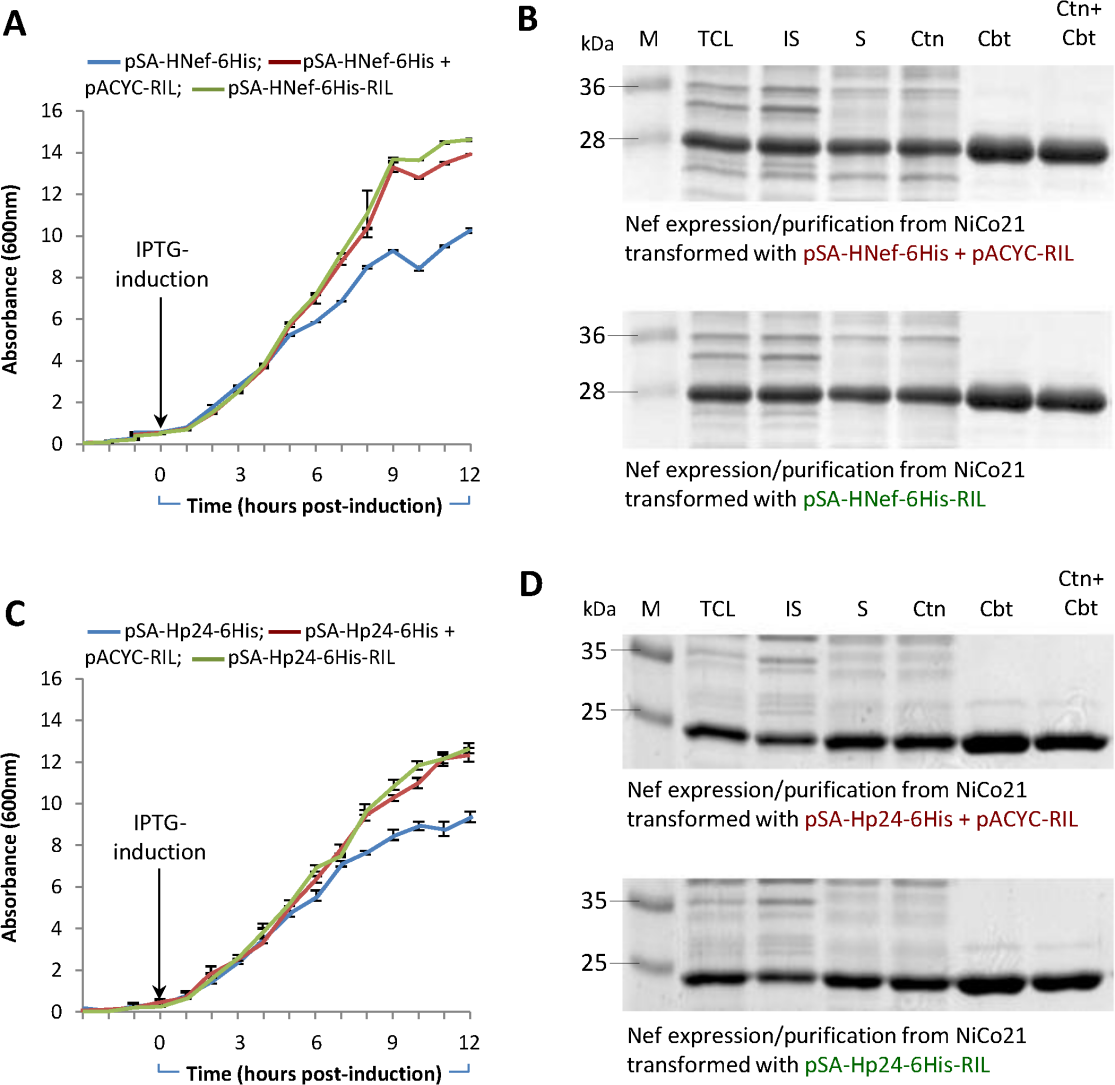
Production and purification of HIV-1 Nef and P24. NiCo21(DE3) transformed with pSA-HNef/P24-6His, pSA-HNef/P24-6His/pACYC-RIL, and pSA-HNef/P24-6His-RIL were grown overnight from a single colony at 30°C in super broth (SB) containing appropriate antibiotic(s). The cultures were then diluted to 0.05 OD_600_ in the same medium and appropriate antibiotics(s). Cultures were grown (at 30°C) to mid-log phase (A600 ~0.5–0.6), at which point IPTG was added to a final concentration of 0.05mM. The induced cells were grown for another 12 hours at 22°C. A small aliquot (100μL) of culture was removed every hour, diluted 10 fold in the same medium and cell density was measured at OD_600_ and plotted. **A.** Growth kinetics of cultures expressing HIV-1 Nef from various expression vectors/combinations. **B.** Comparison of Nef production/purification from cultures expressing Nef from pSA-HNef-6His/pACYC-RIL with those expressing Nef from pSA-HNef-6His-RIL vector. **C.** Growth kinetics of cultures expressing HIV-1 P24 from various expression vectors/combinations. **D.** Comparison of P24 production/purification from cultures expressing P24 from pSA-HP24-6His/pACYC-RIL with those expressing P24 from pSA-HP24-6His-RIL vector.

Bacterial cultures were grown using optimized conditions *i*.*e*. super broth (SB) as culture medium, 0.05mM IPTG as inducer, and cultivation at 22°C for 12h. Cells were harvested and optical densities at 600nm and total biomass yield were determined. Lysed bacterial pellets were used to purify Nef and P24/CA as described in *Materials and Methods*. Growth curves were similar between the cultures expressing Nef or P24/CA from pSA-HNef/P24-6His-RIL and pSA-HNef/P24-6His/pACYC-RIL. Cultures expressing Nef or P24 from pSA-HNef/P24-6His exhibited a lag in growth (**Fig 10A and 10C**) suggestive of Nef and P24 toxicity towards bacteria when overexpressed in the absence of rare tRNAs. Highly purified Nef and P24/CA were obtained from cultures expressing *nef* and *p24* in presence of rare tRNAs (**Fig 10B and 10D**). Using the optimized conditions, we obtained ~5–5.5 mg highly pure (up to 84%) recombinant HV-1 Nef and P24/CA protein from each 1g biomass of pSA-HNef/P24-6His-RIL or pSA-HNef/P24-6His + pACYC-RIL –transformed NiCo21(DE3) bacteria when propagated in SB, and induced with 0.05mM IPTG for 12h at 22°C. There appeared no difference in the final yields and purity of Nef or P24 produced by single (pSA-HNef/P24-6His-RIL) and traditional double-vector (pSA-HNef/P24-6His + pACYC-RIL) system.

## Discussion

We have engineered an expression vector that concomitantly expresses the heterologous protein of interest, and rare tRNA genes in *E*. *coli*. We started off with cloning HIV-1 *nef* gene in an expression vector pSA-HP24-6His, which has previously been used for high level expression of HIV-1 P24 [13]. Nef is an accessory protein of HIV that is responsible for a number of viral pathogenic effects. Progression to AIDS is delayed and in some well-documented cases abolished on infection with naturally occurring HIV strains that contain truncated or mutated *nef* sequences in their genomes [28, 29]. It has been proposed that specific Nef inhibitors could potentially abrogate or slow down gradual deterioration of immune system in chronically infected patients in absence of functional cure [14, 15, 16, 17, 18, 19]. We are engineering therapeutic antibodies against HIV-1 Nef, and therefore need large amounts of highly purified recombinant protein.

Little Nef was produced when expressed in NiCo21(DE3) *E*. *coli*, and optimization of expression conditions had no effect on the final yield. We then analyzed *nef* gene for the presence of codons rarely used in *E*. *coli*, and found that *nef* contained 8.21% of such codons. We therefore anticipated that co-expression of rare tRNA genes from a helper plasmid could help with high level Nef expression. Indeed, almost 8-fold increase in expression was achieved when Nef was produced in the presence of a rare tRNA-expressing helper plasmid (pACYC-RIL). Nef expression is toxic towards expression hosts such as bacteria and yeast [30, 31], though the exact mechanism of this toxicity is not known. We observed that Nef expression was toxic towards *E*. *coli* but only when it was expressed in the absence of rare tRNA genes. We anticipate that this could be due to the misincorporation or omission of one or several rare tRNA-coded arginine and/or lysines, which results in the production of mutant Nef protein(s) that is toxic towards the expression host. It is also possible that rare tRNAs get sequestered by ribosomes engaged in the translation of rare codon –containing heterologous gene that may affect the cell’s ability to produce essential endogenous protein needed for optimal cell growth.

In order to construct a vector that could concomitantly express the *nef* and the rare tRNA genes, we modified the backbone of the resulting pSA-HNef-6His vector by replacing a non-essential DNA segment between *lac*I gene and T7 promoter with *arg*U, *ile*Y, and *leu*W tRNA genes. The resultant vector was called pSA-HNef-6His-RIL, which was then used to produce high levels of recombinant HIV-1 Nef protein. In addition to *nef* gene, we also cloned HIV-1 *p24* and *vif*, two genes that contain 10.77 and 14.5% rare codons respectively. In side-by-side experiments we showed that the *p24* and *vif* genes expressed from single pSA-P24/Vif-6His-RIL vector produced similar or better levels of recombinant proteins compared to traditional two vector system in which rare tRNA genes are expressed from a ColE1 –compatible helper plasmid such as pACYC-RIL.

It would be interesting to note that protein expression in presence of rare tRNA genes yielded two fold more P24 and Vif compared to eight fold more Nef, whereas the *p27 (nef)* gene contains fewer rare codons (8.25% of total codons) compared to *p24* (10.77% of total codons) and *vif* (14.5%) genes. Moreover, HIV-1 *p27(nef)* gene contains rare codons for two amino acids arginine and leucine, whereas *p24* and *vif* genes contain rare codons for four amino acids arginine, leucine, isoleucine, and proline. These observations suggest that neither total number of rare codons nor all the rare codons affect the heterologous protein expression in *E*. *coli*. Upon close examination we noticed four rare codons (the last two as tandem repeat) arranged in a cluster in the 5’ of *p27* (*nef*) gene encoding arginine (highlighted region in **S1A Fig**). There are another two rare codons for arginine also arranged as tandem repeat in the middle of the *nef* gene (highlighted region in **S1A Fig**). We did not notice similar clustering of arginine –encoding rare codons in either *p24* or *vif* genes (**S1B and S1C Fig**). These observations are in agreement with previous findings that not all rare codons affect the heterologous protein expression and it is the priority among those rare codons (for instance those encode for arginine), their position in the gene, and tandem arrangement that affects the protein expression in *E*. *coli* [32].

**Fig 11:**
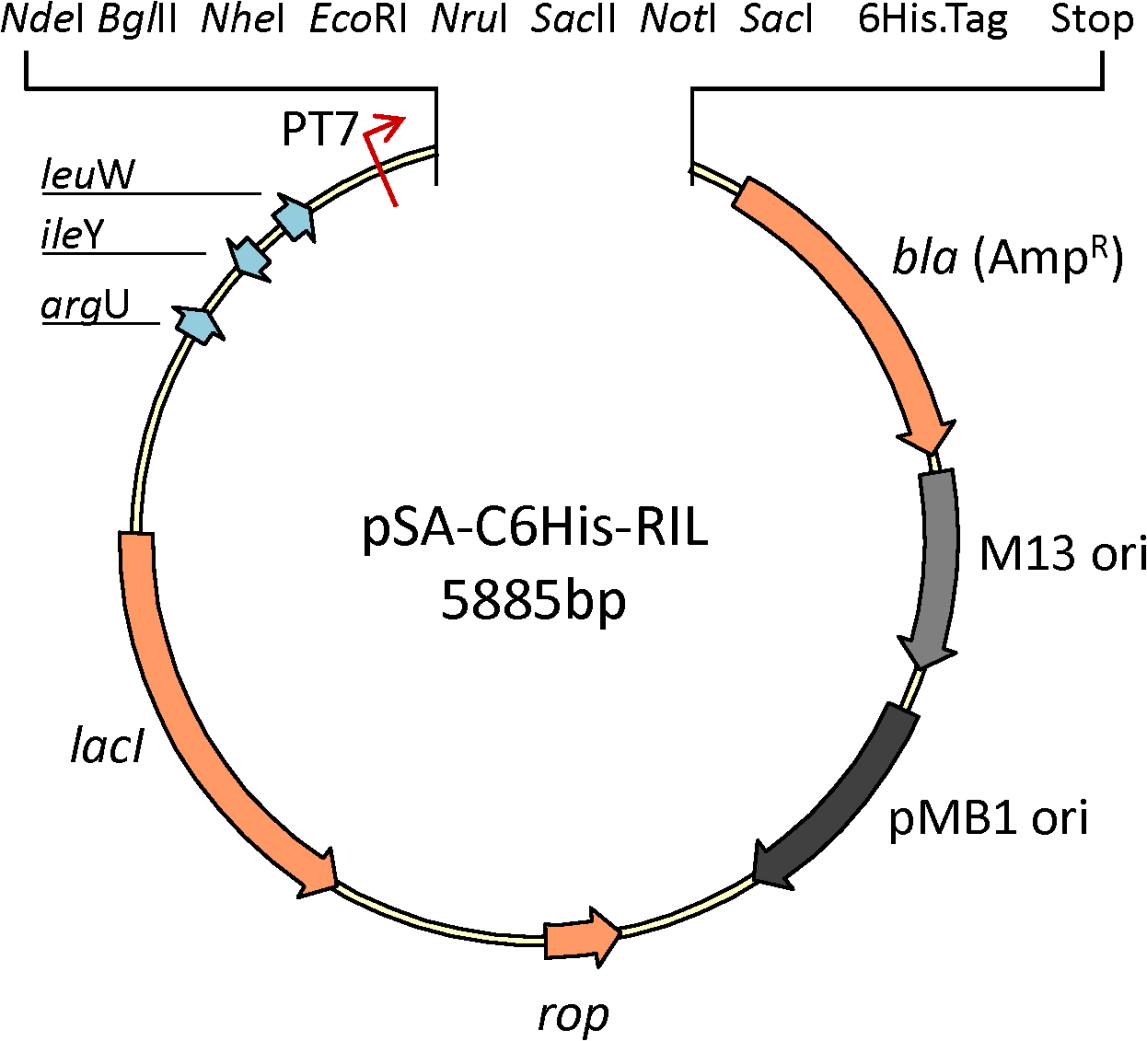
Schematic representation of expression vectors pSA-C6His-RIL. Map shows multiple cloning site with eight unique restriction enzyme sites for facile cloning of heterologous genes.

To facilitate clone manipulation, we substituted *nef* gene with a multiple cloning site (MCS) clustered with eight unique restriction enzyme sites (**Fig 11**). Any two of the restriction enzyme sites can be chosen to clone gene of interest into the pSA-C6His-RIL vector. However it will be desirable to clone the gene of interest between *Nde*I and *Sac*I, in order to avoid the addition of extra amino acids at N- and/or C-terminals of the resultant recombinant protein. Another possibility is to introduce desired restriction enzyme sites to the plasmid using whole-plasmid PCR. Several high fidelity polymerases suitable for whole-plasmid PCR are now available at reasonable cost. Whenever possible, we amplify the entire plasmid backbone with a set of primers containing desired restriction enzyme sites using Phusion or Q5 High-Fidelity DNA Polymerases. By using 50–100 ng plasmid vector as template, and using 18–25 PCR cycles, it is possible to obtain sufficient amount of PCR amplified plasmid, which can be used to clone the gene of interest with compatible restriction enzymes sites. To reduce the background, we add 10U of *Dpn*I restriction enzymes and 1U of alkaline phosphatase in the reaction. This treatment effectively eliminates the bacterially-produced methylated-plasmid template, and dephosphorylates the ends of the restricted PCR amplified plasmid vector.

Eschenfeldt and co-workers have recently described the construction of LIC expression vectors containing rare tRNA genes [33]. These vectors contain tRNA genes covering rare codons for arginine (AGG/AGA) and isoleucine (AUA), and accept heterologous genes via LIC (ligation independent cloning). While these vectors are useful, they are limited in their utility due to the presence of only two rare tRNA genes. Furthermore, albeit of its advantages over restriction enzyme –based cloning, LIC is not a widely used method in various labs. The expression vector described in the present study contains three rare tRNA genes and therefore suitable for the expression of an assortment of heterologous genes in *E*. *coli*. We understand that cloning and expression of heterologous genes in the described rare tRNA-containing expression vector will save both time, and cost, and prove a useful addition to the existing tools/reagents available to scientific community.

## Supporting Information

S1 Fig Rarely used codons in HIV-1 *nef*, *p24*, and *vif* genes. occurrence of rarely used codons in HIV-1 (NL4.3) *nef* (A), *p24* (B) and *vif* (C) genes.
(TIFF)
