# Supplementary figures and images for: Engineering and Validation of a Vector for Concomitant Expression of Rare Transfer RNA (tRNA) and HIV-1 nef Genes in Escherichia coli
Source: PLoS One. 2015 Jul 6;10(7):e0130446. doi: 10.1371/journal.pone.0130446 (PMC4492947; doi:10.1371/journal.pone.0130446)

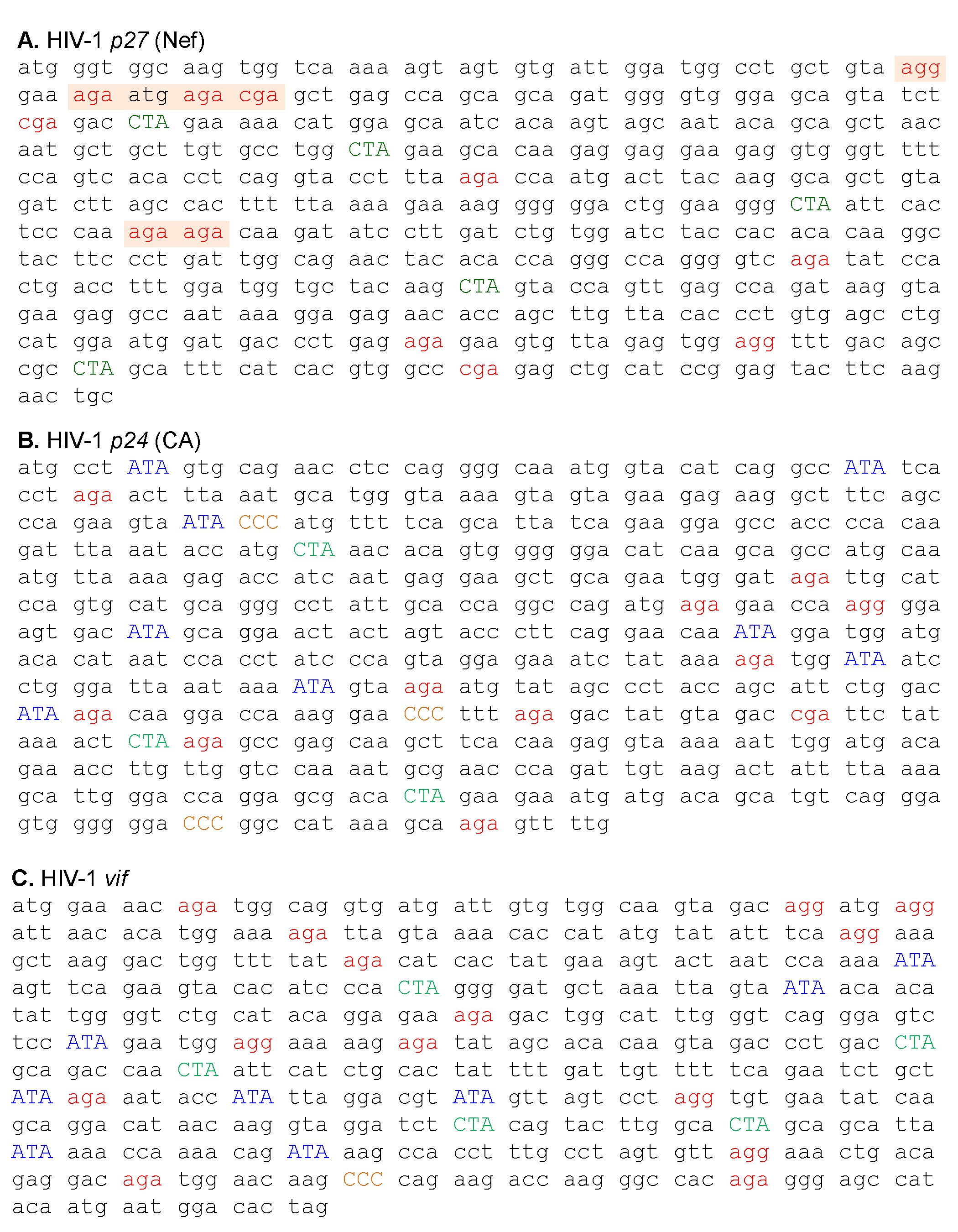

Supplement: S1 Fig — occurrence of rarely used codons in HIV-1 (NL4.3) nef (A), p24 (B) and vif (C) genes. (TIFF) [file pone.0130446.s001.tiff]
